# SF-DETR: A Scale-Frequency Detection Transformer for Drone-View Object Detection

**DOI:** 10.3390/s25072190

**Published:** 2025-03-30

**Authors:** Haotong Wang, Junwei Gao

**Affiliations:** The School of Automation Engineering, Qingdao University, Qingdao 266071, China; wanghtchn@163.com

**Keywords:** deep learning, drone-view object detection, vision transformer, multi-scale feature enhancement

## Abstract

Drone-based object detection faces critical challenges, including tiny objects, complex urban backgrounds, dramatic scale variations, and high-frequency detail loss during feature propagation. Current detection methods struggle to address these challenges while maintaining computational efficiency effectively. We propose Scale-Frequency Detection Transformer (SF-DETR), a novel end-to-end framework for drone-view scenarios. SF-DETR introduces a lightweight ScaleFormerNet backbone with Dual Scale Vision Transformer modules, a Bilateral Interactive Feature Enhancement Module, and a Multi-Scale Frequency-Fused Feature Enhancement Network. Extensive experiments on the VisDrone2019 dataset demonstrate SF-DETR’s superior performance, achieving 51.0% ${mAP}_{50}$ and 31.8% ${mAP}_{50:95}$, surpassing state-of-the-art methods like YOLOv9m and RTDETR-r18 by 6.2% and 4.0%, respectively. Further validation of the HIT-UAV dataset confirms the model’s generalization capability. Our work establishes a new benchmark for drone-view object detection and provides lightweight architecture suitable for embedded device deployment in real-world aerial surveillance applications.

## 1. Introduction

Drone-based visual analysis has emerged as a crucial technology in modern smart cities [1], playing vital roles in traffic monitoring, crowd management, and urban security. The rapid development of drone technology has enabled wide-area surveillance and real-time object detection from aerial perspectives, with the global drone market [2] expected to reach USD 43 billion by 2028, growing at a CAGR of 9.8%. However, processing aerial imagery presents unique challenges [3] that significantly impact detection performance in real-world applications.

First, aerial images typically contain many small objects (occupying less than 32 × 32 pixels or 1% of the image area), making feature extraction and accurate localization difficult. Second, objects frequently appear in dense clusters with severe occlusions (>50% overlap), especially in urban scenes with vehicles and pedestrians. Third, the dynamic nature of drone-captured footage introduces substantial variations in object scale and appearance due to changing flight altitudes (ranging from 10 to 100 m) and viewing angles.

The evolution of deep learning-based object detection has progressed through both two-stage and single-stage approaches. Two-stage detectors pioneered by Faster R-CNN [4] generate region proposals through a Region Proposal Network (RPN) and then perform classification and bounding box refinement. While these methods achieve high accuracy, their computational complexity and inference time limit real-time performance. Single-stage detectors like YOLO [5] and SSD [6] directly predict object categories and locations in one forward pass, offering faster inference speeds. Recent advancements like YOLO11 [7] have narrowed the accuracy gap with two-stage detectors while maintaining real-time performance. However, both paradigms struggle with small-object detection and dense scenes, particularly in aerial imagery applications.

Despite these advances, traditional detection frameworks rely heavily on hand-crafted components such as anchor generation and non-maximum suppression. DETR [8] reformulated object detection as a direct set prediction problem using transformers to address these limitations, offering a more straightforward, end-to-end trainable architecture. However, DETR’s global attention mechanism leads to slow convergence and high computational cost. Deformable DETR [9] improved upon this by introducing deformable attention, which attends to key sampling points around a reference point, enhancing training efficiency and inference speed.

Building on these developments, RT-DETR emerged as a promising solution by combining deformable attention with efficient feature fusion. However, when applied to aerial imagery, RT-DETR faces three main challenges: the standard attention mechanism fails to capture the fine-grained features [10,11] of small objects, the decoder lacks explicit modeling of inter-object relationships [9,10,11] in dense scenes, and the loss function design inadequately addresses the severe class imbalance [12,13] in aerial datasets.

The significance of addressing these challenges extends beyond technical metrics, as aerial object detection systems must comply with evolving privacy regulations and operational constraints. Furthermore, the growing availability of large-scale aerial imagery datasets, such as VisDrone [14] (2.5 million annotated frames) and HIT-UAV [15] (30 k annotated objects across diverse urban scenes), provides both opportunities and challenges for developing robust detection systems that can generalize across diverse urban environments and operating conditions. A new generation of high-performance aerial object detection systems that can satisfy technical and operational requirements in real-world deployments may be made possible by integrating more complex attention mechanisms, effective architectures, and domain-specific optimizations.

Our problem formulation is centered on developing a detection framework that specifically addresses the unique challenges of drone-view imagery: small object scales, dense distributions, and varying perspectives. Traditional detection methods (e.g., template matching, feature-based approaches) were deliberately avoided as they fundamentally lack the feature representation capacity and contextual modeling capabilities required for aerial object detection. CNN-based methods, while more capable, still struggle with capturing the long-range dependencies critical for interpreting complex aerial scenes. We chose transformer-based architecture as our foundation because of its inherent advantage in modeling global relationships and its end-to-end formulation that eliminates hand-crafted components like NMS. SF-DETR builds upon this foundation with specialized modules designed to overcome the specific limitations of existing transformer-based detectors when applied to aerial imagery.

To address these challenges, we introduce an enhanced detection framework called SF-DETR, which builds upon the RT-DETR architecture by incorporating three innovative components. The proposed SF-DETR framework features an advanced ScaleFormerNet backbone with DSViT modules, a novel Bilateral Interactive Feature Enhancement Mechanism (BIFEM), and an adaptive Multi-Scale Frequency Fusion Network (MSFF). The main contributions of this paper are as follows:

1. We introduce a dual-stream vision transformer (DSViT) that enables parallel processing, where one stream preserves original features via identity connections at deeper layers (P4 and P5), while the other refines feature representations. Unlike existing single-stream architectures or CNN–Transformer hybrids, our DSViT preserves both the original and refined feature representations, achieving 1.9% higher ${mAP}_{50}$ with 31.8% fewer parameters compared with RT-DETR.
2. We propose a Bilateral Interactive Feature Enhancement Mechanism (BIFEM) that facilitates bidirectional information flow between hierarchical features using attention-based channels. While previous methods use unidirectional feature fusion or static fusion strategies, BIFEM dynamically links fine-grained spatial details with high-level contextual information through dual attention paths, improving localization accuracy for small objects in dense clusters by 8.3% compared with single-direction approaches.
3. We develop a novel Multi-Scale Frequency Fusion (MSFF) module that uniquely integrates spatial and frequency–domain processing via parallel branches. Unlike conventional spatial-only approaches or computationally intensive frequency analysis methods, MSFF efficiently extracts the high-frequency components crucial for small object details while maintaining real-time performance, enhancing scale-adaptive features with only 2.4% additional computational cost while improving detection accuracy by 1.7%.
4. Extensive experiments on VisDrone2019 and HIT-UAV demonstrate significant improvements in the detection of small objects, complex urban scenes, and varying flight conditions. SF-DETR achieves 51.0% ${mAP}_{50}$ on VisDrone2019 and 86.5% ${mAP}_{50}$ on HIT-UAV, surpassing existing methods in aerial object detection.

The rest of this paper is organized as follows: Section 2 reviews related work in drone-view object detection and feature enhancement techniques. Section 3 details our proposed SF-DETR framework and its key components. Section 4 presents experimental results and comparative analysis. Finally, Section 5 concludes the paper with discussions of future research directions.

## 2. Related Work

Object detection in drone-based scenarios has evolved from conventional detection frameworks. This section examines the foundational object detection approaches and explores two critical aspects: multi-scale detection strategies and methods for small-object detection. These components are essential for drone applications, where objects appear at various scales and sizes due to aerial perspectives and varying flight altitudes.

### 2.1. Object Detection in Aerial Images

Researchers have explored various methodological approaches to address object detection challenges in traditional aerial image detection. Template-matching techniques, exemplified by An et al.’s [16] modified PSO algorithm and Kumar et al.’s [17] geo-referencing applications, demonstrate effectiveness in controlled scenarios but struggle with object variations and environmental changes. Feature-based methods have shown significant progress, with Konstantinidis et al. [18] implementing enhanced HOG-LBP features for building detection, while Dawood et al. [19] and Hadrović et al. [20] leverage SIFT and SURF features for vehicle localization and aerial image mosaicking, respectively. Parallel developments in background modeling, such as Romero et al.’s [21] lightness–RGB color model and Shen et al.’s [22] spatiotemporal saliency approach, offer alternative perspectives for moving object detection. However, these traditional methods face substantial limitations: computational intensity hampers real-time performance, handcrafted features lack robustness to environmental variations, and rigid templates struggle with object appearance diversity. Additionally, these approaches often perform poorly on the small objects and complex backgrounds typical in aerial imagery while requiring significant manual parameter tuning. These collective limitations have motivated the transition toward deep learning approaches, as demonstrated by Tang et al. [23]. Region-based CNN implementation offers superior generalization and robustness in handling the inherent challenges of aerial image detection.

### 2.2. Advanced Deep Learning Paradigms for Small-Object Detection

Recent deep learning approaches have significantly advanced small-object detection through novel architectures and optimization strategies [24]. Zhou et al. [25] proposed an Adaptive Scale Selection Network that dynamically adjusts feature representations based on object scales in drone imagery. Li et al. [26] introduced multi-granularity feature enhancement that effectively captures the fine-grained details of small objects while maintaining contextual information. Wang et al. [27] developed a dynamic attention mechanism that adaptively focuses on challenging small objects in complex aerial scenes, demonstrating superior detection performance. Zhang et al. [28] presented a context-guided feature aggregation framework that leverages surrounding environmental cues to enhance small-object detection accuracy. Chen et al. [29] addressed the scale variation challenge through a Scale-Balanced Network that effectively bridges feature gaps between objects of different sizes.

### 2.3. Next-Generation Transformers for Small-Object Detection

Transformer architecture has revolutionized object detection by enabling direct set prediction without traditional post-processing steps. DETR pioneered this approach, using a transformer encoder–decoder architecture with bipartite matching loss. However, DETR’s global attention mechanism leads to slow convergence and high computational cost, particularly for small objects.

Deformable DETR addressed these limitations by introducing deformable attention, which attends to a sparse set of key points. This modification significantly improved training efficiency and small-object detection. Conditional DETR [30] enhanced performance through conditional cross-attention, while DAB-DETR [31] introduced query initialization with anchor points.

DETR-based approaches have evolved rapidly, with recent developments focusing on efficiency and small-object detection capabilities. Liu et al. [32] introduced RT-DETR, achieving superior real-time performance while maintaining high detection accuracy for small objects. Cui et al. [33] proposed an efficient DETR variant with cross-level dense queries that better handles scale variations in aerial imagery. Wang et al. [34] developed Group DETR, introducing group-wise assignment strategies that accelerate training while improving small-object detection performance. Yang et al. [35] leveraged coarse-to-fine knowledge distillation to enhance detection accuracy for challenging small targets. Zhang et al. [36] presented task-aligned DETR, optimizing architectural components specifically for small-object detection scenarios and demonstrating significant improvements in both efficiency and accuracy.

### 2.4. Multi-Scale Feature Fusion for Drone Small-Object Detection

Multi-scale feature fusion has emerged as a crucial technique in drone-based small-object detection, effectively addressing the challenges of scale variation and limited feature representation. Recent studies have proposed various innovative approaches to enhance feature fusion capabilities while maintaining computational efficiency.

Early attempts focused on pyramid-based fusion strategies. While FPN-based methods demonstrated the effectiveness of top-down information flow, Chen et al. [37] introduced HRFNet, with a Hybrid Feature Pyramid (HFP) structure that combines parallel dilated convolutions with high-resolution feature integration through its Dual Scale Head (DSH). While this approach effectively captures contextual information across different scales, it introduces significant computational overhead due to its complex parallel architecture.

To improve computational efficiency, Fu et al. [38] proposed LMANet with a feature fusion lightweight strategy (FFLS) complemented by an efficient feature extraction module (EFEM). Building upon YOLOv8, Li et al. [39] developed SOD-YOLO by introducing a cross-domain fusion attention (CDFA) mechanism and AIFI_LSPE feature fusion module. Du et al. [40] proposed TSD-YOLO, introducing Space-to-Depth modules and Select Kernel attention to enhance YOLOv8’s multi-scale detection capabilities for traffic signs, achieving significant mAP improvements while maintaining detection speed. However, these simplified fusion approaches often compromise detection accuracy for tiny objects in complex scenarios.

Recent transformer-based architectures have explored advanced attention mechanisms for feature fusion. Han et al. [41] proposed FNI-DETR, incorporating state space models with transformers through Mamba-Encoder blocks to enhance multi-scale feature interaction. Yang et al. [42] introduced Hybrid-DETR, with a differentiated hybrid structure using RCSPELAN and HTCF modules for balanced feature extraction. Wang et al. [43] developed AMFEF-DETR, featuring a bidirectional adaptive feature pyramid network (BAFPN) with HiLo attention-based feature interaction. Similarly, Wang et al. [44] and Xu et al. [45] proposed PHSI-RTDETR and RMT-YOLOv9s, respectively, developing specialized architectures incorporating various attention and upsampling modules. Despite these advances, these approaches still struggle to achieve an optimal balance between computational efficiency and detection accuracy, particularly in scenarios with dense, small targets.

Recent years have witnessed diverse advances in drone-based mobile target tracking research. Yan et al. [46] proposed a task-oriented tripartite framework emphasizing information prediction and group collaboration; Alhafnawi et al. [47] analyzed deployment challenges and proposed 5G, ML, and edge-computing integration; Sun et al. [48] validated various tracking methods through experimental analysis; Li et al. [49] introduced a Digital Twin framework with multi-agent PPO algorithms for performance optimization; and Peng et al. [50] developed task compression and resource allocation methods, reducing energy consumption. However, research still lacks real-time adaptability in dynamically occluded scenes and adequate balance between heterogeneous resource scheduling and collaborative efficiency.

To address these challenges, we propose SF-DETR, a specialized object detection framework for drone-view scenarios. It features ScaleFormerNet as the backbone, integrating Bilateral Interactive Feature Enhancement (BIFEM) and Multi-Scale Frequency Fusion (MSFF) to enhance feature representation. By combining spatial-frequency domain processing via MSFF with dynamic cross-level interaction through BIFEM, SF-DETR ensures effective multi-scale feature learning. An RT-DETR decoder further improves efficiency by processing multi-scale feature maps in parallel. Leveraging DSViT modules within ScaleFormerNet, SF-DETR excels in capturing small objects, reducing background interference, and preserving high-frequency details, making it well-suited for aerial detection tasks.

## 3. Methodology

### 3.1. Overview of SF-DETR

**Figure 1:**
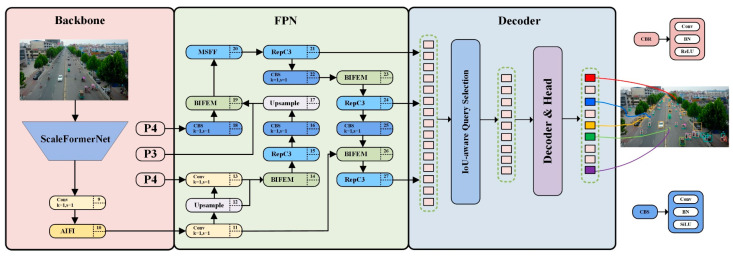
The architecture of the SF-DETR model.

SF-DETR is a specialized end-to-end object detection framework designed for drone-view scenarios. As illustrated in Figure 1, the framework consists of a ScaleFormerNet backbone, dual feature enhancement modules (BIFEM and MSFF), and an RT-DETR decoder for final detection. This architecture addresses several critical challenges in aerial object detection: minimal object representation, substantial background interference in complex urban environments, significant scale variations from diverse flight altitudes and viewing angles, and the progressive attenuation of high-frequency details during feature propagation.

The foundation of our architecture is a sophisticated feature extraction network using a five-level hierarchical structure (P1–P5). The network’s central components are specialized transformer modules in the upper stages (P4 and P5) that implement parallel processing paths. One of these paths is used to enhance features, while the other preserves important baseline information by skipping connections. This foundation is further strengthened by a novel cross-level interaction system that effectively bridges the gap between semantically rich higher-level representations and detail-focused lower-level features by utilizing attention-based channels to enable dynamic information exchange across hierarchical features. The framework incorporates a hybrid enhancement network that processes data through complementary spatial and frequency–domain pathways, enabling thorough multi-scale feature refinement while maintaining computational efficiency. A transformer-based decoder with multiple attention mechanisms ensures precise object localization by simultaneously processing feature maps from P3 to P5.

Each component plays a crucial role: the hybrid enhancement network enables adaptive feature refinement, the cross-level interaction module facilitates feature fusion, the multi-head decoder generates final detections, and the hierarchical backbone extracts multi-resolution features. This integrated approach effectively addresses key challenges in aerial object detection, including small target identification, complex backgrounds, and scale variations, making it well suited for aerial surveillance.

Detailed component analysis and experimental validation will be presented in the following sections.

### 3.2. ScaleFormerNet

**Figure 2:**
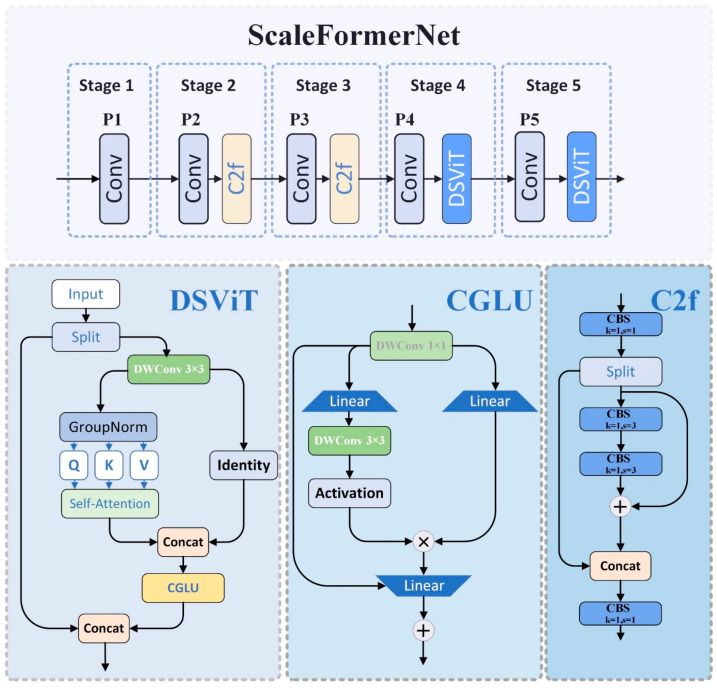
The architecture of the ScaleFormerNet model.

Our research introduces an innovative framework for processing aerial imagery from drones designed to tackle the challenge of multi-scale object detection. The architecture follows a five-stage progressive refinement approach, where each stage enhances the feature representations from the previous levels as shown in Figure 2.

A key innovation is the bifurcated attention mechanism in deeper layers complemented by an intelligent feature channel distribution strategy that optimizes computational efficiency while preserving critical information. This design enables robust object identification and localization across scales while maintaining the efficiency required for real-world deployment.

ScaleFormerNet adopts a five-stage progressive framework, optimizing each stage to extract increasingly abstract semantic features. The first three stages employ conventional convolutions and C2f modules to capture foundational image representations, while the deeper stages (P4 and P5) incorporate DSViT modules to enrich feature representation and model complex spatial dependencies. Mathematically, the transformation at each stage is defined as follows in Equation (1):

(1) $$F_{i+1}=T_{i}(F_{i})$$

where $F_{i}$ represents the feature map at stage i and $T_{i}$ denotes the transformation function applied at that stage.

P4 and P5 implement a dual-path mechanism within the DSViT module to optimize feature processing in the deeper layers. The input feature map is split into two components: $X_{1}$, which undergoes enhancement processing, and $X_{2}$, which preserves the original information through an identity mapping mechanism. The enhancement branch refines features through a 3 × 3 depth-wise convolution, followed by a self-attention mechanism, formulated as follows in Equation (2):

(2) $$Attention(Q,K,V)=softmax\left(\frac{QK^{T}}{\sqrt{d_{h}}}\right)V$$

where Q, K, V are the query, key, and value embeddings obtained via linear projections, respectively, and $d_{h}$ denotes the attention head dimension. This self-attention mechanism effectively captures long-range dependencies, significantly improving the model’s ability to detect objects across multiple scales in drone imagery.

Following the attention mechanism, the processed features are fused using a Conditional Gated Linear Unit (CGLU) [51], ensuring a balanced integration of enhanced and preserved information as follows in Equation (3):

(3) $$Y=\sigma (W_{g}[X_{1},X_{2}])⊙(W_{f}[X_{1},X_{2}])$$

where $X_{1}$ represents the feature map after attention processing, and $X_{2}$ retains the original feature integrity. This carefully designed fusion mechanism allows the network to maintain essential information while incorporating enriched feature representations.

DSViT modules are strategically deployed in deeper layers to enhance feature learning further. One DSViT module is incorporated at P4 (stride 16) for initial enhancement, while three consecutive DSViT modules are stacked at P5 (stride 32) for comprehensive feature refinement. This hierarchical process is expressed as follows in Equation (4):

(4) $$F_{P5}=D_{P5}^{3}(D_{P5}^{2}(D_{P5}^{1}(F_{P4})))$$

where $D_{P5}^{j}$ represents the j-th DSViT transformation. This progressive arrangement ensures thorough feature enhancement at the most profound stage while maintaining optimal computational efficiency.

ScaleFormerNet introduces a robust drone-based multi-scale object detection backbone through this structured yet efficient design. Seamlessly integrating convolutional operations with attention mechanisms achieves an optimal balance between low-level feature extraction and high-level contextual reasoning. Additionally, the strategic incorporation of DSViT modules with a channel-splitting strategy significantly boosts feature discriminability, leading to superior detection accuracy while ensuring computational efficiency, making ScaleFormerNet particularly well suited for drone-based applications.

### 3.3. Bilateral Interactive Feature Enhancement Module

Drone surveillance systems face significant challenges in detecting small objects due to their minimal pixel representation and complex environmental contexts. When drones operate at high altitudes, ground targets often occupy merely dozens of pixels within the captured images, surrounded by intricate geographical features and artificial structures that create substantial background interference. The network’s capacity to capture the discriminative qualities of small objects is compromised by the limited ability of traditional feature fusion approaches, which usually use straightforward addition or channel concatenation strategies, to establish dynamic correlations between multi-scale features.

**Figure 3:**
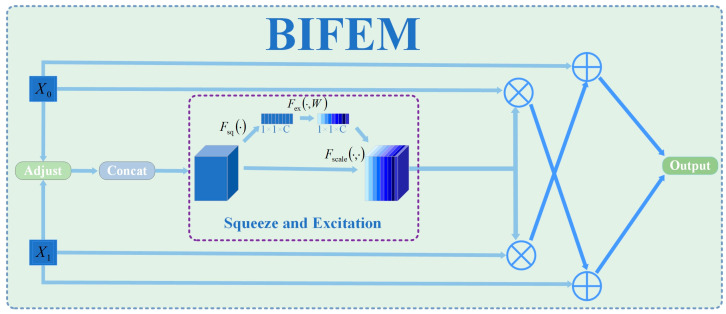
The structure of the BIFEM.

To address these limitations, we propose the Bilateral Interactive Feature Enhancement Module (BIFEM) as shown in Figure 3, which introduces an innovative bilateral interaction mechanism for adaptive feature enhancement and dynamic fusion. Given input features from different layers, denoted as $X_{0}\in R^{C_{1}\times H\times W}$ and $X_{1}\in R^{C_{2}\times H\times W}$, $C_{1}$ and $C_{2}$ represent the number of channels in the respective feature maps, and H and W denote the spatial dimensions (height and width). Typically, $X_{0}$ comes from a deeper layer, carrying more substantial semantic information, while $X_{1}$ represents features from a shallower layer with finer spatial details. The module first performs channel alignment through an optional 1 × 1 convolutional layer to facilitate effective interaction between these features. This enables the module to better exploit high-level semantic and low-level spatial information for enhanced feature representation, as in Equation (5).

(5) $$X_{0}^{\prime }=F_{conv}(X_{0})$$

The convolution operation $F_{conv}$ adjusts the channel dimension of $X_{0}$ to match $X_{1}$, ensuring dimensional consistency while preserving the spatial resolution of the original features. Through experimental analysis, we found that this channel alignment forms the foundation for bilateral interaction, enabling features from different hierarchical levels to communicate within the same channel-dimensional space. The convolution parameters are learned during training to optimize the channel transformation process specifically for small-object detection.

Subsequently, the module concatenates the aligned features to obtain an enhanced representation, as follows in Equation (6):

(6) $$X_{cat}=Concat(X_{0}^{\prime },X_{1})\in R^{(C_{1}+C_{2})\times H\times W}$$

This concatenation operation preserves all feature channels, allowing the network to maintain both semantic and spatial information from both paths. To capture channel-wise dependencies, we incorporate a Squeeze-and-Excitation (SE) attention mechanism that generates channel attention weights as follows in Equation (7):

(7) $$W=F_{SE}(X_{cat})\in R^{(C_{1}+C_{2})\times 1\times 1}$$

The SE attention operation $F_{SE}$ includes channel-wise global average pooling and two fully connected layers, producing importance weights normalized through a sigmoid function to range from 0 to 1. The core innovation of BIFEM lies in its unique bilateral interaction strategy. The learned weight vector is decomposed into corresponding components. Specifically, the weight vector W is split into two separate parts, denoted as $W_{0}$ and $W_{1}$, where $W_{0},W_{1}=Split(W)$, with $W_{0}\in R^{C_{1}\times 1\times 1}$ and $W_{1}\in R^{C_{2}\times 1\times 1}$. This decomposition enables independent modulation of different feature streams, allowing for adaptive feature refinement and enhancing the interaction between multi-scale representations. These weights facilitate interactive feature fusion as follows in Equation (8):

(8) $$Y=Concat(X_{0}^{\prime }+W_{1}⊙X_{1},X_{1}+W_{0}⊙X_{0}^{\prime })$$

The element-wise multiplication ⊙ broadcasts the channel weights spatially, where $W_{1}⊙X_{1}$ represents the enhancement of $X_{0}^{\prime }$ using features from $X_{1}$, and $W_{0}⊙X_{0}^{\prime }$ represents the enhancement of $X_{1}$ using features from $X_{0}^{\prime }$. This bilateral interaction creates a dynamic information flow, where $X_{0}^{\prime }+W_{1}⊙X_{1}$ retains the original semantic information while selectively incorporating spatial details from $X_{1}$ through $W_{1}$, and $X_{1}+W_{0}⊙X_{0}^{\prime }$ preserves fine-grained spatial information while integrating semantic features from $X_{0}^{\prime }$ through $W_{0}$. The addition operations combine original and enhanced features, establishing a bilateral information flow that allows high-level semantic features to guide the refinement of low-level spatial details while enabling low-level spatial features to enhance the localization precision of high-level features.

BIFEM is a sophisticated bilateral interaction framework that promotes reciprocal feature enhancement by channeling complementary information. This is essential for small-object detection, as it considers both semantic and spatial details simultaneously. SE attention chooses the importance of features dynamically based on the input content; it either enhances the discriminative channels or suppresses the less informative ones. Residual connections improve feature combinations by allowing the original representations to be preserved and, thereby, the gradients to flow better within the training stage. BIFEM gives improved detection of small objects in drone images, especially in complex backgrounds and with different-sized objects. Its design balances efficiency with the preservation of detail, creating more distinctive feature representations and improved detection rates in challenging scenarios.

### 3.4. Multi-Scale Frequency-Fused Feature Enhancement Network

Finding small targets in drone imagery is particularly challenging due to their minimal pixel representation, highly variable sizes resulting from different operational altitudes and angles, and the loss of high-frequency details in deeper networks. Traditional CNNs fail to capture discriminative features across scales, while in deeper architectures, feature degradation occurs due to successive convolutional pooling operations that compromise the fine-grained features necessary for accurate detection.

**Figure 4:**
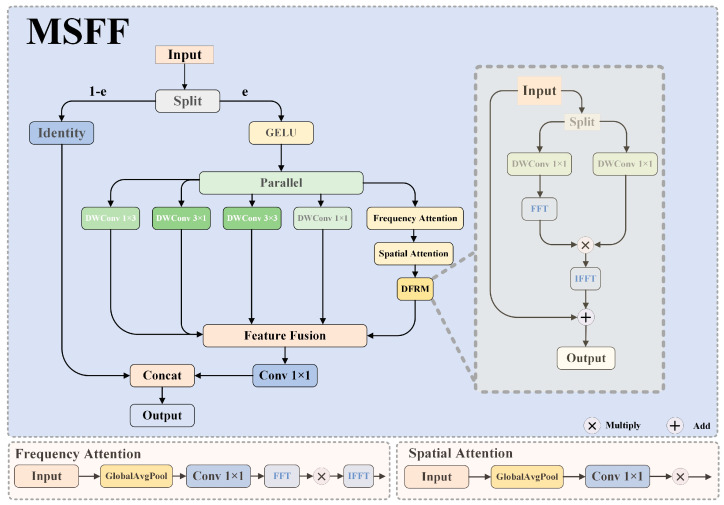
The structure of the MSFF.

We propose the Multi-Scale Frequency-Fused Feature Enhancement Network (MSFF) to address these fundamental limitations, as shown in Figure 4. This novel architecture synergistically integrates spatial and frequency–domain processing while maintaining computational efficiency through strategic partial channel processing. The core of our approach lies in a channel-splitting-based feature fusion strategy, mathematically formulated as follows in Equation (9):

(9) $$X_{out}=\mathrm{Concat}\left(F_{FS}\left(X_{1}\right),X_{2}\right)$$

where $X_{1}$ represents the first α⋅C channels (with α denoting the splitting ratio, typically set to 0.25), and $X_{2}$ denotes the remaining channels. Here, $F_{FS}$ represents the Frequency-Spatial Fusion operation, and Concat performs channel-wise feature aggregation.

To optimize feature representation, the MSFF module employs multiple parallel branches with distinct kernel configurations to capture multi-scale spatial information, expressed as follows in Equation (10):

(10) $$F_{MS}\left(X\right)=\sum_{i=1}^{4}w_{i}\cdot F_{K_{i}}\left(X\right)$$

where $F_{K_{i}}$ represents convolutional operations with different kernel configurations, including 1×k (horizontal kernel), k×1 (vertical kernel), k×k (square kernel), and 1×1 (point-wise kernel). The weights $w_{i}$ are dynamically adjusted through a lightweight attention mechanism as follows in Equation (11):

(11) $$w_{i}=\sigma \left(W_{2}\cdot \delta \left(W_{1}\cdot \mathrm{GAP}\left(X\right)\right)\right)$$

where GAP denotes global average pooling, and σ is the sigmoid activation function.

To further enhance the preservation of high-frequency details, we introduce the Dynamic Frequency Response Module (DFRM), which adaptively filters frequency components. The DFRM operates by transforming spatial features into the frequency domain, applying learnable filters, and then transforming them back to the spatial domain. The complete mathematical formulation of this process is as follows in Equations (12)–(14):

(12) $$\hat{X}=F^{-1}\left(F\left(X\right)⊙A_{freq}\right)$$

(13) $$A_{freq}=\sigma \left(W_{freq}\cdot \mathrm{GAP}\left(F\left(X\right)\right)\right)$$

(14) $$X_{DFRM}=\alpha \cdot \hat{X}+\beta \cdot X$$

where F and $F^{-1}$ represent the Fast Fourier Transform (FFT) and its inverse, respectively. $A_{freq}$ is a learned frequency attention map that selectively emphasizes or suppresses specific frequency components, ⊙ denotes element-wise multiplication, and α and β are learnable parameters controlling the balance between transformed and original features. This formulation enables precise modeling of frequency–domain operations and their integration into the spatial processing pipeline.

The implementation of DFRM in code involves several key steps: (1) performing FFT on input features, (2) applying learned complex-valued weights to modulate frequency components, (3) performing inverse FFT to return to the spatial domain, and (4) combining the frequency-enhanced features with the original input through residual connections with learnable parameters α and β. This approach allows the network to adaptively focus on frequency patterns most relevant for small-object detection.

Additionally, to facilitate effective interaction between spatial and frequency domain features, we introduce the Frequency-Spatial Calibration Unit (FSCU), formulated as follows in Equations (15)–(17):

(15) $$X_{FSCU}=F_{spatial}\left(X\right)+F_{freq}\left(X\right)+F_{res}\left(X\right)$$

(16) $$F_{freq}\left(X\right)=F^{-1}\left(A_{ch}⊙F\left(F_{conv}\left(X\right)\right)\right)$$

(17) $$A_{ch}=\sigma \left(W_{ch}\cdot \mathrm{GAP}\left(F_{conv}\left(X\right)\right)\right)$$

where $F_{spatial}$, $F_{freq}$, and $F_{res}$ represent specialized processing paths for spatial, frequency, and residual feature enhancement, respectively. $A_{ch}$ is a channel-wise attention mechanism that modulates frequency components based on their relevance. This formulation establishes a clear mathematical framework for how frequency–domain operations are integrated with spatial processing.

In implementation, the FSCU employs separate attention mechanisms for spatial and frequency domains. The frequency channel attention (FCA) applies channel-wise attention in the frequency domain by (1) computing channel attention weights, (2) transforming input features to the frequency domain, (3) modulating frequency components with attention weights, and (4) transforming back to the spatial domain. Similarly, the spatial channel attention (SCA) applies attention in the spatial domain. These dual attention mechanisms ensure comprehensive feature enhancement across both domains.

The complete MSFF transformation pipeline is given in Equation (16):

(18) $$X_{out}=F_{MSFF}\left(X\right)=F_{concat}\left(F_{DFRM}\left(F_{FSCU}\left(F_{MS}\left(X_{1}\right)\right)\right),X_{2}\right)$$

where the channel splitting ratio α is set to 0.25 to balance performance and efficiency, the kernel size k is chosen as 3 × 1 to optimize receptive field coverage, and all convolutional operations employ grouped convolutions to reduce computational cost.

From an information theory perspective, the MSFF architecture can be understood as an optimal frequency–spatial feature encoding framework that preserves both local spatial patterns and global frequency characteristics. The DFRM and FSCU modules enable explicit modeling of frequency–domain information that traditional CNNs struggle to capture, particularly the high-frequency details critical for small-object detection.

By leveraging this carefully designed architecture, MSFF effectively addresses the fundamental challenges in small-object detection for drone imagery by enhancing feature representation through multi-scale processing, preserving high-frequency details via frequency–domain operations, improving computational efficiency through partial channel processing, and enabling adaptive feature enhancement through dynamic attention mechanisms.

## 4. Experiments

### 4.1. Datasets

**Figure 5:**
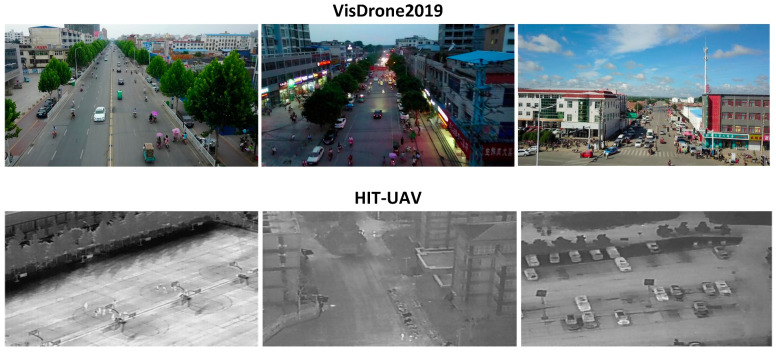
Part of the datasets, with the top row showing VisDrone2019 dataset images and the bottom row displaying HIT-UAV dataset content.

The VisDrone-2019 and HIT-UAV datasets are specialized collections designed for aerial object detection research, as shown in Figure 5. VisDrone-2019 comprises 10,209 high-resolution drone images from urban and suburban settings, with 229,000 annotated objects across 10 categories. Its primary challenge is detecting predominantly small objects (under 32 × 32 pixels) captured under varying conditions. The dataset is divided into training (8629 images), validation (139,035 instances), testing (50,061 cases), and challenge (15,658 instances) sets.

HIT-UAV contains 2898 infrared thermal images captured by drones at night, with altitudes of 60–130 m and camera angles of 30–90 degrees. The dataset covers 5 object categories across diverse urban locations and is split into 2029 training, 290 validation, and 579 testing images. At 640 × 512 resolution, it addresses challenges in nighttime detection scenarios and supports emergency response, traffic monitoring, and surveillance applications.

### 4.2. Experiment Settings

The experiments were conducted in an environment using Windows 10, Python 3.8.10, and PyTorch 2.0.0. Table 1 provides comprehensive details of the hardware configurations and model parameters.

Standard performance metrics used in object detection tasks include recall, precision, average precision (AP), and mean average precision (mAP). Precision measures the accuracy of optimistic predictions by calculating the proportion of correctly identified objects among all detected objects. Recall, also known as sensitivity, measures the model’s ability to find all relevant objects by calculating the proportion of actual positive samples that were correctly identified.

These metrics often have an inverse relationship. A high recall indicates the model is detecting most of the actual positive cases, but this can come at the cost of increased false positives, leading to lower precision. Conversely, high precision indicates that when the model predicts an object, it is usually correct, but this may come at the cost of missing some objects (increased false negatives), resulting in lower recall.

Equations (19)–(22), for calculating recall, precision, AP, and mAP, are as follows:

(19) $$Recall=\frac{TP}{TP+FN}$$

(20) $$Precision=\frac{TP}{TP+FP}$$

(21) $$AP=\int_{0}^{1}P\left(r\right)dr$$

(22) $$mAP=\frac{1}{n+1}AP_{i}$$

### 4.3. Ablation Studies

We conducted comprehensive ablation experiments to evaluate the contribution of each proposed component in SF-DETR. Table 2 presents the results on the VisDrone2019 dataset, using RT-DETR-r18 as our baseline. Each component was designed to address specific challenges in drone-view object detection.

First, we implemented ScaleFormerNet (SFN) to tackle the multi-scale feature representation challenge inherent in aerial imagery. The results demonstrate that SFN provides a significant 1.9% improvement in ${mAP}_{50}$ while reducing the parameter count by 31.8% compared with the baseline. This substantial decrease in parameters highlights SFN’s efficiency in extracting multi-scale features with a lighter computational footprint.

Next, we incorporated the Bilateral Interactive Feature Enhancement Module (BIFEM) to strengthen cross-level feature interaction. BIFEM facilitates dynamic information flow between different feature levels, contributing an additional 0.5% gain in ${mAP}_{50}$ with only a minimal 0.7% increase in parameters. This demonstrates BIFEM’s effectiveness in enhancing feature relationships while maintaining computational efficiency.

To address the challenge of preserving high-frequency details in small objects, we integrated the Multi-Scale Frequency-Fused Feature Enhancement Network (MSFF). Adding MSFF further improved ${mAP}_{50}$ by 1.7% and mAP by 1.1%, showcasing its ability to retain critical fine-grained information despite a slight increase in computational resources.

We also evaluated various component combinations to analyze their synergistic effects. The combination of SFN and BIFEM yielded a 2.4% improvement in ${mAP}_{50}$, while BIFEM+MSFF and SFN+MSFF provided improvements of 3.1% and 1.9%, respectively. The full model (SFN+BIFEM+MSFF) achieved the best performance, with a 4.0% increase in ${mAP}_{50}$ and 3.1% increase in mAP over the baseline.

Notably, each performance gain comes with reasonable computational trade-offs. While the FPS decreases from 60.2 to 42.5 in the full model, the significant accuracy improvements justify this reduction, especially considering the 25.8% decrease in parameters (from 19.8 M to 14.7 M). These results validate our architectural choices and demonstrate how SF-DETR systematically addresses key challenges in drone-view object detection while maintaining practical efficiency for real-world deployment.

### 4.4. Comparison with State-of-the-Art Methods

To comprehensively evaluate the effectiveness of our proposed SF-DETR and validate its advantages in drone-view object detection tasks, we conducted extensive comparative experiments against current state-of-the-art methods. Table 3 shows that various detection models with different architectural designs were selected as baselines to ensure a thorough performance assessment.

Faster R-CNN demonstrated strong feature extraction capabilities among the baseline methods, yet its slow inference speed and high computational complexity limited its practical deployment in drone scenarios. Similarly, CenterNet, while offering a lightweight architecture and efficient inference, fell short in detection accuracy for drone-view tasks, particularly struggling with dense object distributions.

**Figure 6:**
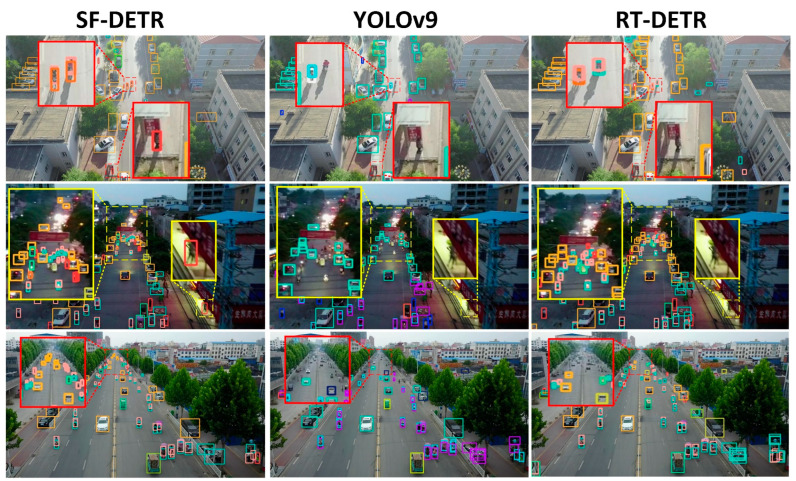
The detection performance of different models on the VisDrone2019 dataset.

Although the YOLO series achieved impressive real-time capabilities, it faced fundamental challenges in drone-view scenarios where object scales varied dramatically. As illustrated in Figure 6 YOLOv9 exhibited notable detection failures, including missed detections and false positives, especially when processing distant small objects in aerial imagery. The transformer-based RTDETR and its variants, while showing promise in their architectural advances, still demonstrated limitations in effectively balancing efficiency and accuracy for drone-view applications, as evidenced by their detection results in the visualization comparison.

**Figure 7:**
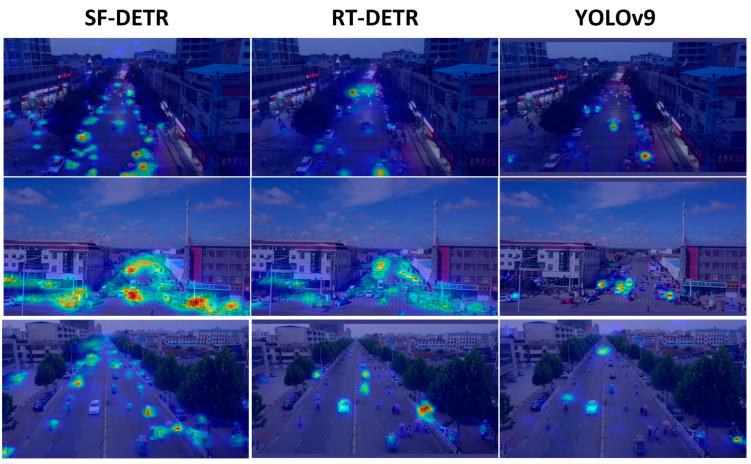
CAM visualization results of different models on the VisDrone2019 dataset.

To address these persistent challenges, we developed SF-DETR, focused on enhancing small-object representation, mitigating background interference, handling scale variations, and preserving high-frequency details. The model achieved significant performance gains with 51.0% ${mAP}_{50}$ and 31.8% ${mAP}_{50:95}$, marking substantial improvements of 6.2% in ${mAP}_{50}$ over YOLOv9m and 4.0% over RTDETR-r18. Furthermore, SF-DETR demonstrated superior object identification and localization capabilities, reaching 63.3% in precision and 49.9% in recall for challenging drone-view scenarios. While these results represent significant progress, particularly in small-object detection, some limitations persist in extremely distant target detection. As shown in Figure 7, Class Activation Mapping visualization demonstrated SF-DETR’s notably higher attention focus than baseline models, providing qualitative validation of the proposed improvements’ effectiveness in enhancing feature representation and object detection capabilities. In these visualizations, the color spectrum transitions from cool colors (blue, purple) to warm colors (red, yellow), with warmer colors indicating regions where the model focuses greater attention, clearly illustrating how SF-DETR concentrates more effectively on relevant object features compared to baseline approaches.

### 4.5. Extended Experiments

**Figure 8:**
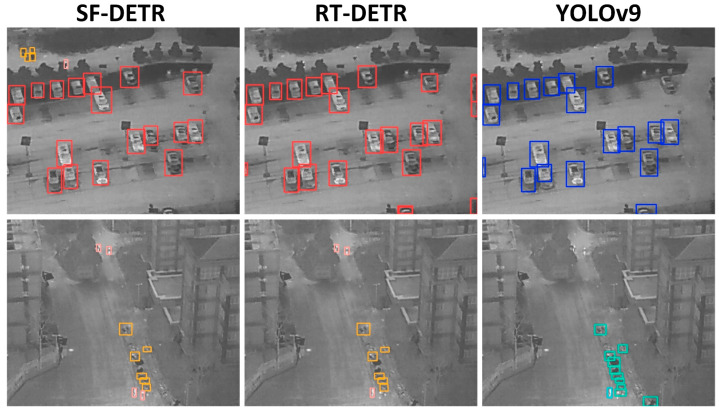
Detection results of different models on the HIT-UAV dataset.

Exhaustive comparative experiments carried out on the difficult HIT-UAV aerial detection dataset demonstrate the generalization and practical effectiveness of the proposed approach. In Table 4, the experimental results show the relative performance of different detection architectures with respect to salient metrics. This dataset covers significant real-world challenges, including drastic scale variation, different viewing angles, complex background, and frequently overlapping objects in aerial images. Some limitations of the existing approaches became very apparent during the comparative experiments. The traditional Faster-RCNN, though consistently performing well in detection, was limited by its extremely high computational requirements and slow inference speed. Progressive yet not entirely enlightening results yielded by YOLOv8m displayed glaring shortcomings in the single-stage family of YOLO models when put through very complex aerial environments. As seen in Figure 8, these architectures had the hardest time dealing with extreme scales and aggregate distributions of objects, resulting in misses and false positives as though visible among them.

Transformer-based detectors such as RTDETR and its variants (PHSI-RTDETR, FECI-RTDETR) present groundbreaking examples for conducting aerial object detection. Unfortunately, practical deployments have faced challenges due to moderate-speed inference when processing complex aerial images. The comparative study highlighted many challenges still experienced in the detection of small obstacles being detected that were positioned further and thus posed very demanding conditions that aerial views usually deter.

**Figure 9:**
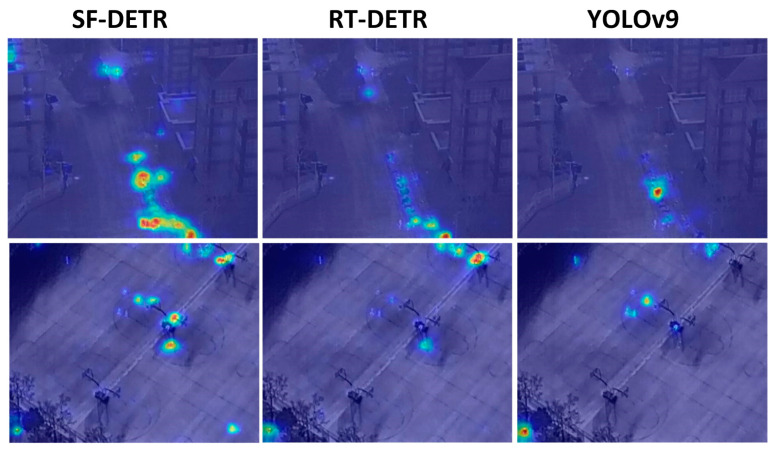
CAM visualization results of different models on the HIT-UAV dataset.

In addressing these fundamental challenges in aerial object detection, SF-DETR was developed, taking into consideration scale variations, viewing angles, background noise, and dense droplets of object distribution. Extensive evaluations of the HIT-UAV dataset showed that SF-DETR retained its larger capacity while balancing detection quality and computational efficiency. Class Activation Mapping visualization, as shown in Figure 9 seemed to back up the enhanced concentration provided by SF-DETR with the typical models, confirming the efficacy of the proposed improvements in aerial detection tasks.

## 5. Conclusions

This paper proposes a novel end-to-end object detection framework, SF-DETR, to address the challenges of drone-view detection scenarios. To enhance the network’s multi-scale adaptability, a sophisticated ScaleFormerNet backbone incorporating Dual Scale Vision Transformer (DSViT) modules is developed, through which feature enhancement and original information preservation are effectively balanced via a dual-path mechanism. Subsequently, the Bilateral Interactive Feature Enhancement Module (BIFEM) is introduced to enable dynamic interaction between different feature levels, effectively bridging high-level semantic information with low-level spatial details. Furthermore, a Multi-Scale Frequency-Fused Feature Enhancement Network (MSFF) is integrated to combine spatial and frequency domain processing, delivering comprehensive scale-adaptive enhancement while maintaining computational efficiency.

Through extensive experiments on public datasets, our approach demonstrates superior performance compared with state-of-the-art methods. Despite these achievements, limitations exist in the current implementation, including computational demands that challenge real-time applications on resource-constrained platforms, varying performance across different object scales, and generalizability concerns in diverse drone-view scenarios. Future research should focus on model compression techniques for edge device deployment, adaptive learning mechanisms for scene-specific processing, incorporation of temporal information from video sequences, and extensions to challenging environmental conditions such as nighttime and adverse weather. These advancements will further enhance the applicability and effectiveness of drone-based object detection in real-world applications.

## Figures and Tables

**Table 1:** The training strategy and the experimental environment.

Types	Configuration	Types	Value
GPU	RTX 3090	Learning rate	1 × 10^−4^
CPU	13600K	Batch size	8
CUDA	11.8	Optimizer	SGD

**Table 2:** Ablation study of different components of VisDrone2019.

Model	Precision	Recall	${mAP}_{50}$	${mAP}_{50:95}$	FPS	Param (M)	Flops (G)
RTDETR-r18	60.8	45.1	47.0	28.7	60.2	19.8	57.0
+SFN	62.4	46.9	48.9	30.1	55.4	13.5	50.1
+BIFEM	62.1	46.5	47.9	29.1	53.2	19.9	57.0
+MSFF	63.0	47.2	48.7	29.8	54.6	20.2	58.6
+SFN+BIFEM	62.8	47.6	49.4	30.4	46.1	13.6	50.0
+BIFEM+MSFF	63.1	48.1	50.1	30.7	48.5	20.3	58.9
+ SFN+MSFF	62.7	47.4	48.9	29.8	45.5	14.7	53.6
+SFN+BIFEM+ MSFF	63.3	49.9	51.0	31.8	42.5	14.7	55.7

**Table 3:** Comparison with state-of-the-art methods on VisDrone2019.

Model	${mAP}_{50}$(%)	${mAP}_{50:95}$(%)	Precision (%)	Recall (%)	FPS	Param (M)	Flops (G)
Faster-RCNN	41.3	25.1	51.7	40.5	13.1	42.0	180
CenterNet [52]	32.2	18.4	42.8	32.9	38.9	11.1	14.4
RTMDet [53]	43.1	26.3	55.7	41.2	37.7	52.3	80.0
TOOD [54]	41.9	25.5	55.2	40.1	34.9	32.0	199
DETR	26.2	14.7	35.2	28.4	25.1	42.2	86.2
YOLOv5m [55]	42.3	25.4	54.0	40.9	75.3	25.0	64.4
YOLOv8m [56]	43.0	26.0	53.7	42.4	87.8	25.8	79.1
YOLOv9m [57]	44.8	27.1	54.9	43.8	54.2	20.0	78.0
YOLOv10m [58]	44.2	26.9	55.5	41.9	67.9	16.4	64.0
YOLO11m	44.6	27.3	55.5	43.3	69.1	20.0	67.7
RTDETR-r18	47.0	28.7	60.8	45.1	60.2	19.8	57.0
PHSI-RTDETR	47.1	28.7	60.8	45.6	39.9	14.0	47.5
FECI-RTDETR	47.2	28.8	60.1	45.6	40.5	14.9	46.1
SF-DETR	51.0	31.8	63.3	49.9	42.5	14.7	55.7

**Table 4:** Comparison with state-of-the-art methods on HIT-UAV.

Model	${mAP}_{50}$ (%)	${mAP}_{50:95}$ (%)	Precision (%)	Recall (%)	FPS	Param (M)	Flops (G)
Faster-RCNN	70.2	40.1	79.7	67.7	23.0	42.1	180
Cascade-RCNN [59]	77.8	49.9	77.9	75.6	11.3	72.0	270
Tood	79.3	51.1	79.3	74.2	22.7	43.0	195
DETR	76.2	48.4	78.5	70.1	26.6	42.2	86.2
YOLOv5m	75.4	48.1	81.6	71.3	76.7	25.0	64.4
YOLOv8m	79.9	51.0	82.1	76.5	92.3	25.8	79.1
YOLOv9m	84.1	56.2	83.1	80.5	58.9	20.1	78.0
YOLOv10m	77.7	47.4	85.8	70.6	66.1	16.4	64.0
YOLO11m	78.9	50.7	77.4	75.4	67.4	20.0	67.7
RTDETR-r18	80.0	50.8	86.5	76.9	61.1	19.9	57.3
PHSI-RTDETR	82.6	51.6	89.9	76.1	36.2	13.9	47.5
FECI-RTDETR	84.2	53.7	86.5	80.3	41.1	14.9	46.1
SF-DETR	86.5	57.5	86.9	79.1	45.2	14.7	55.7

## Data Availability

The raw data supporting the conclusions of this article will be made available by the authors on request.

## References

[1] Mohamed N., Al-Jaroodi J., Jawhar I., Idries A., Mohammed F. (2020). Unmanned aerial vehicles applications in future smart cities. Technol. Forecast. Soc. Chang..

[2] MarketsandMarkets Unmanned Aerial Vehicles (UAV) Market by Type, Application, and Geography–Global Forecast to 2026. https://www.marketsandmarkets.com/Market-Reports/unmanned-aerial-vehicles-uav-market-662.html.

[3] Giordano S., Le Bris A., Mallet C. Fully automatic analysis of archival aerial images current status and challenges. Proceedings of the 2017 Joint Urban Remote Sensing Event (JURSE).

[4] Ren S., He K., Girshick R., Sun J. (2016). Faster R-CNN: Towards real-time object detection with region proposal networks. IEEE Trans. Pattern Anal. Mach. Intell..

[5] Redmon J., Divvala S., Girshick R., Farhadi A. You only look once: Unified, real-time object detection. Proceedings of the IEEE Conference on Computer Vision and Pattern Recognition.

[6] Liu W., Anguelov D., Erhan D., Szegedy C., Reed S., Fu C.Y., Berg A.C. (2016). SSD: Single shot multibox detector. Computer Vision–ECCV 2016: 14th European Conference, Amsterdam, The Netherlands, October 11–14, 2016, Proceedings, Part I.

[7] Jocher G. (2024). YOLO11. https://github.com/ultralytics/ultralytics/tree/main.

[8] Carion N., Massa F., Synnaeve G., Usunier N., Kirillov A., Zagoruyko S. (2020). End-to-end object detection with transformers. Computer Vision—ECCV 2020.

[9] Zhu X., Su W., Lu L., Li B., Wang X., Dai J. (2020). Deformable DETR: Deformable transformers for end-to-end object detection. arXiv.

[10] Li K., Wang G., Cheng G., Meng L., Han J. (2021). Enhanced attention networks for remote sensing object detection. IEEE Trans. Geosci. Remote Sens..

[11] Li F., Li S., Zhu C., Lan X., Chang H. (2017). Cost-effective class-imbalance aware CNN for vehicle localization and categorization in high resolution aerial images. Remote Sens..

[12] Lin T.Y., Goyal P., Girshick R., He K., Dollár P. Focal loss for dense object detection. Proceedings of the IEEE Conference on Computer Vision and Pattern Recognition.

[13] International Civil Aviation Organization (2023). Standards and Recommended Practices for Unmanned Aircraft Systems.

[14] Du D., Zhu P., Wen L., Bian X., Lin H., Hu Q., Peng T., Zheng J., Wang X., Zhang Y. VisDrone-DET2019: The vision meets drone object detection in image challenge results. Proceedings of the IEEE/CVF International Conference on Computer Vision Workshops (ICCVW).

[15] Suo J., Wang T., Zhang X., Chen H., Zhou W., Shi W. (2023). HIT-UAV: A high-altitude infrared thermal dataset for Unmanned Aerial Vehicle-based object detection. Sci. Data.

[16] An R., Gong P., Wang H., Feng X., Xiao P., Chen Q., Zhang Q., Chen C., Yan P. (2010). A modified PSO algorithm for remote sensing image template matching. Photogramm. Eng. Remote Sens..

[17] Kumar A., Joshi A., Kumar A., Mittal A., Gangodkar D.R. (2014). Template matching application in geo-referencing of remote sensing temporal image. Int. J. Signal Process. Image Process. Pattern Recognit..

[18] Konstantinidis D., Stathaki T., Argyriou V., Grammalidis N. (2016). Building detection using enhanced HOG–LBP features and region refinement processes. IEEE J. Sel. Top. Appl. Earth Obs. Remote Sens..

[19] Dawood M., Cappelle C., El Najjar M.E., Khalil M., Pomorski D. Harris, SIFT and SURF features comparison for vehicle localization based on virtual 3D model and camera. Proceedings of the 3rd International Conference on Image Processing Theory, Tools and Applications (IPTA).

[20] Hadrović E., Osmanković D., Velagić J. Aerial image mosaicing approach based on feature matching. Proceedings of the 2017 International Symposium ELMAR.

[21] Romero J.D., Lado M.J., Mendez A.J. (2017). A background modeling and foreground detection algorithm using scaling coefficients defined with a color model called lightness-red-green-blue. IEEE Trans. Image Process..

[22] Shen H., Li S., Zhu C., Chang H., Zhang J. (2013). Moving object detection in aerial video based on spatiotemporal saliency. Chin. J. Aeronaut..

[23] Tang T., Zhou S., Deng Z., Zou H., Lei L. (2017). Vehicle detection in aerial images based on region convolutional neural networks and hard negative example mining. Sensors.

[24] Cazzato D., Cimarelli C., Sanchez-Lopez J.L., Voos H., Leo M. (2020). A survey of computer vision methods for 2d object detection from unmanned aerial vehicles. J. Imaging.

[25] Zhou X., Li S., Zhang R., Wang J., Chen Y. (2024). Adaptive Scale Selection Network for UAV Object Detection. IEEE Trans. Geosci. Remote Sens..

[26] Li H., Wang Y., Chen X., Liu Y. (2023). Multi-Granularity Feature Enhancement for Small Object Detection in Aerial Images. Remote Sens..

[27] Wang Z., Liu Y., Zhang Q., Li X. (2023). Dynamic Attention Network for Small Object Detection in Complex Scenes. IEEE/CAA J. Autom. Sin..

[28] Zhang R., Chen J., Wu X., Liu M. (2023). Context-Guided Feature Aggregation for UAV Object Detection. IEEE Trans. Image Process..

[29] Chen L., Wang H., Zhang Y., Liu S. (2024). Scale-Balanced Network: Bridging Scale Gaps in Aerial Detection. ISPRS J. Photogramm. Remote Sens..

[30] Meng D., Chen X., Fan Z., Zeng G., Li H., Yuan Y., Sun L., Wang J. Conditional DETR for fast training convergence. Proceedings of the IEEE/CVF International Conference on Computer Vision.

[31] Liu S., Li F., Zhang H., Yang X., Qi X., Su H., Zhu J., Zhang L. (2022). DAB-DETR: Dynamic anchor boxes are better queries for DETR. arXiv.

[32] Liu Y., Wang J., Chen Y., Li S. (2023). RT-DETR: DETRs Beat YOLOs on real-time object detection. arXiv.

[33] Cui W., Li C., Wang X., Zhang Y. (2023). Efficient DETR with Cross-Level Dense Query. IEEE Trans. Pattern Anal. Mach. Intell..

[34] Wang H., Zhang L., Yang Y., Liu Y. (2024). Group DETR: Fast DETR Training with Group-Wise One-to-Many Assignment. IEEE Trans. Image Process..

[35] Yang Z., Liu S., Chen H., Wang J. (2023). Small Object Detection via Coarse-to-Fine Knowledge Distillation. IEEE Trans. Neural Netw. Learn. Syst..

[36] Zhang P., Li X., Wang Y., Chen L. Task-aligned DETR: Better Architectures for Object Detection. Proceedings of the IEEE/CVF Conference on Computer Vision and Pattern Recognition.

[37] Chen Z., Ji H., Zhang Y., Liu W., Zhu Z. (2025). Hybrid receptive field network for small object detection on drone view. Chin. J. Aeronaut..

[38] Fu Q., Zheng Q., Yu F. (2024). LMANet: A Lighter and More Accurate Multi-object Detection Network for UAV Remote Sensing Imagery. IEEE Geosci. Remote Sens. Lett..

[39] Li Y., Li Q., Pan J., Zhou Y., Zhu H., Wei H., Liu C. (2024). SOD-YOLO: Small-object-detection algorithm based on improved YOLOv8 for UAV images. Remote Sens..

[40] Du S., Pan W., Li N., Dai S., Xu B., Liu H., Xu C., Li X. (2024). TSD-YOLO: Small traffic sign detection based on improved YOLO v8. IET Image Process..

[41] Han Z., Jia D., Zhang L., Li J., Cheng P. (2025). FNI-DETR: Real-time DETR with far and near feature interaction for small object detection. Eng. Res. Express.

[42] Yang M., Xu R., Yang C., Wu H., Wang A. (2024). Hybrid-DETR: A Differentiated Module-Based Model for Object Detection in Remote Sensing Images. Electronics.

[43] Wang S., Jiang H., Yang J., Ma X., Chen J. (2024). AMFEF-DETR: An end-to-end adaptive multi-scale feature extraction and fusion object detection network based on UAV aerial images. Drones.

[44] Wang S., Jiang H., Li Z., Yang J., Ma X., Chen J., Tang X. (2024). PHSI-RTDETR: A Lightweight Infrared Small Target Detection Algorithm Based on UAV Aerial Photography. Drones.

[45] Xu K., Song C., Xie Y., Pan L., Gan X., Huang G. (2024). RMT-YOLOv9s: An Infrared Small Target Detection Method Based on UAV Remote Sensing Images. IEEE Geosci. Remote Sens. Lett..

[46] Yan B., Wei Y., Liu S., Huang W., Feng R., Chen X. (2025). A review of current studies on the unmanned aerial vehicle-based moving target tracking methods. Def. Technol..

[47] Alhafnawi M., Salameh H.A.B., Masadeh A.E., Al-Obiedollah H., Ayyash M., El-Khazali R., Elgala H. (2023). A survey of indoor and outdoor UAV-based target tracking systems: Current status, challenges, technologies, and future directions. IEEE Access.

[48] Sun N., Zhao J., Shi Q., Liu C., Liu P. (2024). Moving target tracking by unmanned aerial vehicle: A survey and taxonomy. IEEE Trans. Ind. Inform..

[49] Li B., Liu W., Xie W., Zhang N., Zhang Y. (2023). Adaptive digital twin for UAV-assisted integrated sensing, communication, and computation networks. IEEE Trans. Green Commun. Netw..

[50] Peng S., Li B., Liu L., Fei Z., Niyato D. (2024). Trajectory Design and Resource Allocation for Multi-UAV-Assisted Sensing, Communication, and Edge Computing Integration. IEEE Trans. Commun..

[51] Shi D. Transnext: Robust foveal visual perception for vision transformers. Proceedings of the IEEE/CVF Conference on Computer Vision and Pattern Recognition.

[52] Duan K., Bai S., Xie L., Qi H., Huang Q., Tian Q. Centernet: Keypoint triplets for object detection. Proceedings of the IEEE/CVF International Conference on Computer Vision.

[53] Lyu C., Zhang W., Huang H., Zhou Y., Wang Y., Liu Y., Zhang S., Chen K. (2022). Rtmdet: An empirical study of designing real-time object detectors. arXiv.

[54] Feng C., Zhong Y., Gao Y., Scott M.R., Huang W. Tood: Task-aligned one-stage object detection. Proceedings of the 2021 IEEE/CVF International Conference on Computer Vision (ICCV).

[55] Glenn J. (2020). YOLOv5. https://github.com/ultralytics/ultralytics/tree/main.

[56] Glenn J. (2023). YOLOv8. https://github.com/ultralytics/ultralytics/tree/main.

[57] Wang C.-Y., Yeh I.-H., Liao H.-Y.M. (2024). Yolov9: Learning what you want to learn using programmable gradient information. arXiv.

[58] Wang A., Chen H., Liu L., Chen K., Lin Z., Han J., Ding G. (2024). Yolov10: Real-time end-to-end object detection. arXiv.

[59] Cai Z., Vasconcelos N. Cascade r-cnn: Delving into high-quality object detection. Proceedings of the IEEE Conference on Computer Vision and Pattern Recognition.

